# Role of Senescent Astrocytes in Health and Disease

**DOI:** 10.3390/ijms24108498

**Published:** 2023-05-09

**Authors:** Jacopo Meldolesi

**Affiliations:** 1 San Raffaele Institute, Vita-Salute San Raffaele University, 20132 Milan, Italy; meldolesi.jacopo@hsr.it; 2 CNR Institute of Neuroscience, Milano-Bicocca University, Vedano al Lambro, 20854 Milan, Italy

**Keywords:** aging, astrocyte heterogeneity, astrocyte reprogramming, cognitive decline, glioblastoma, neurodegeneration, neuroinflammation, senescent astrocytes, tauopathies, up and downregulation

## Abstract

For many decades after their discovery, astrocytes, the abundant glial cells of the brain, were believed to work as a glue, supporting the structure and metabolic functions of neurons. A revolution that started over 30 years ago revealed many additional functions of these cells, including neurogenesis, gliosecretion, glutamate homeostasis, assembly and function of synapses, neuronal metabolism with energy production, and others. These properties have been confirmed, limited however, to proliferating astrocytes. During their aging or following severe brain stress lesions, proliferating astrocytes are converted into their no-longer-proliferating, senescent forms, similar in their morphology but profoundly modified in their functions. The changed specificity of senescent astrocytes is largely due to their altered gene expression. The ensuing effects include downregulation of many properties typical of proliferating astrocytes, and upregulation of many others, concerned with neuroinflammation, release of pro-inflammatory cytokines, dysfunction of synapses, etc., specific to their senescence program. The ensuing decrease in neuronal support and protection by astrocytes induces the development, in vulnerable brain regions, of neuronal toxicity together with cognitive decline. Similar changes, ultimately reinforced by astrocyte aging, are also induced by traumatic events and molecules involved in dynamic processes. Senescent astrocytes play critical roles in the development of many severe brain diseases. The first demonstration, obtained for Alzheimer’s disease less than 10 years ago, contributed to the elimination of the previously predominant neuro-centric amyloid hypothesis. The initial astrocyte effects, operating a considerable time before the appearance of known Alzheimer’s symptoms evolve with the severity of the disease up to their proliferation during the final outcome. Involvement of astrocytes in other neurodegenerative diseases and cancer is now intensely investigated.

## 1. Introduction

Astrocytes, a family of glial cells abundant in the brain, were first recognized in the innovative images produced by Santiago Ramon y Cajal at the end of the 19th century, i.e., over 120 years ago. During the following decades, intensely proliferating astrocytes were believed to operate “as a glue”, a multicellular complex providing adjacent neurons with structural support, molecular exchanges and metabolic processes. The end of the glue concept occurred towards the end of 1990 with the recognition of astrocytes’ involvement in tripartite synapses [1,2] and in gliosecretion, a conventional form of neurosecretion [3]. Almost 30 years ago, the complex of these and other specialized functions were defined as “an astrocyte revolution” [4], an explanation still employed to emphasize further discoveries about astrocytes (see, for example [5]).

Since then, additional roles of proliferating astrocytes have been progressively recognized. The most exciting example is the epigenetic “reprogramming” capability of astrocytes to become neurons. This process occurs naturally in vivo and also experimentally upon astrocyte exposure to exogenous factors such as various injuries. In the adult brain the other well-known neurogenic cells, neural progenitors, are localized in a few specific areas where neurogenesis takes place. In order to reach their appropriate localization, therefore, neurons generated from progenitors need to undergo significant travel. In contrast, reprogramming of astrocytes occurs in many areas where their neurogenesis is regulated by inflammatory signals [6,7,8].

Examples of discovered astrocyte functions deal with synaptogenesis, production and release of trophic factors, glutamate homeostasis, integration into highly specific neuro-glial networks, structure and regulation of blood–brain barrier (BBB) and others [9,10]. Together, these functions—in some cases astrocyte-specific—are critical for the development and general activities of the brain. In other cases, their functions are induced in parallel with or by microglia. In this review, focused on specific aspects of astrocytes, the interactions and co-operations with microglia are not illustrated but only mentioned.

In the central nervous system (CNS), neuronal assistance and protection predominates among the physiological functions of astrocytes. However, these functions do not cover all the properties of these cells. In fact, during aging and also during various forms of severe stress, proliferative astrocytes are converted into non-proliferative senescent astrocytes. Moreover, in the CNS, senescent astrocytes play roles in many complex processes, often associated with the appearance or strong reinforcement of brain diseases. In other words, conversion of astrocyte functions and reactivity contribute markedly to aging of the brain and, over time, to the development of neurodegenerative diseases [11,12,13].

Studies of the last few decades have progressively revealed the processes governing the development of astrocyte senescence starting with arrest of proliferation together with stimulation of pro-inflammatory profiles [11,12,13]. During aging many brain-expressed genes of astrocytes, including those involved in neuronal development and differentiation, are downregulated. Some others, including those associated with senescence—often named senescence-associated secretory phenotype (SASP)—are upregulated [11,12]. Suppression of senescence cell characteristics, such as SASP, as well as caloric restrictions, reduce astrocyte aging [14,15]. Recently, an additional mechanism of aging has been found to depend on autophagy, a dynamic form of cytoplasmic organelle inducing accumulation of altered molecules and structures destined to be degraded upon autophagic fusion with lysosomes. The positive importance of autophagy during aging, revealed in various types of cells [16], has been confirmed and expanded in astrocytes and includes their interaction with neurons [17,18].

For decades the mechanisms governing the interactions of astrocytes with neurons and other brain cells remained mysterious. Over 30 years ago, however, the discovery of exosomes and ectosomes, two types of cytoplasmic vesicles which are released to the extracellular medium and have actions on target cells, has led to the identification of many effects, especially relevant to brain cells. These developments have been extensively investigated, and the ensuing findings have been reported in several reviews, including my own [19,20]. In view of the existing ample knowledge about exosomes and ectosomes, their back-and-forth communications between astrocytes and neurons have been omitted from this review. The other key properties of astrocyte–neuron communication are presented in three Sections focused primarily on major issues of the present review. Section 2 and Section 3 illustrate in detail the properties and mechanisms of action typical of senescent astrocytes including their profound differences with respect to the early proliferating astrocytes. The roles of senescent astrocytes in various types of diseases are presented in Section 4, focused primarily on Alzheimer’s (AD), the most extensively investigated disease. The discovery of the complex effects of senescent astrocytes in this disease has led to the development of general information, valid in pathology combined with concepts of medical/clinical relevance. The Final Comments, starting from representations of the two major issues, reported in the three Sections, summarize the potential interest of senescent astrocytes for therapy, including examples, unclear at present, that are expected to be clarified in the near future.

## 2. Astrocyte Senescence Development from Young Physiology

This Section is focused primarily on senescent astrocytes, converted from the early proliferating forms of these cells by profound changes in their mechanisms and functions. The analogous effects in microglia, and their observed co-operation with astrocytes, are mentioned here only when important for the senescence of the latter cells. 

### 2.1. Physiology of Early Astrocytes

**Figure 1:**
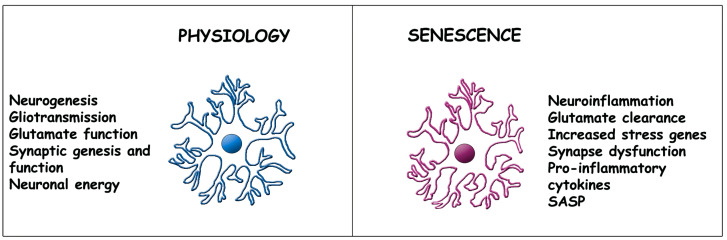
Comparison of structure and function in early vs. senescent astrocytes. The structure of astrocytes during physiology (**left**) is not severely changed upon aging, except for the moderately increased thickness of the appendages (**right**), with at least partial maintenance of their contacts to adjacent cells (not shown). The marked differences concern functions listed on the left and right sides. The state of senescent astrocytes depends primarily on changes in their gene expression, specifically on downregulation of genes governing physiology (examples to the **left**) and upregulation of genes governing new functions or increasing their activity during and upon aging (examples to the **right**). The present astrocyte images are analogous to those of [23].

Early in their activity, these astrocytes contribute to the dynamic balance of the brain governed by a number of functions, some of which were already mentioned in the Introduction (Figure 1) [8,9,10,11,12]. Additional properties and their development during aging are presented here [21]. For many decades, astrocytes were believed to be homogeneous, i.e., expressing all the same structures and functions. Currently, these cells are known to be heterogeneous, composed of several types distinct in structure, function and distribution in the various areas of the brain. Such heterogeneity becomes more and more important during aging, the development of senescence and in various diseases [22]. More detailed presentations are therefore given in two areas, in Section 2.2 and Section 4.

Early astrocytes (left in Figure 1) are critical for the generation and activity of synapses, due to their direct participation, typical of tripartite synapses (1,2). Gliotransmission, a secretory conventional activity of considerable relevance in brain physiology [3,4], occurs by exocytic discharge of various transmitters including glutamate, ATP and the specific D-serine and eicosanoids [4,9,21] released in response to Ca^2+^ oscillations [24]. Astrocyte energy from glucose, lipids and amino acids supports marked neuronal metabolism [25]. Active trophic factors are released from astrocytes by exocytosis and also by activation of plasma membrane channels [21]. Upon their release, these factors are primarily targeted to neurons, inducing protection and axonal regeneration. Feedback and feedforward signaling effects induced by early astrocytes can ultimately tune the balance of neurons between excitation and inhibition [11]. Other cells, including types of glia and blood vessels, are also targeted by astrocytes, however, less frequently [20,21,22,24].

### 2.2. Astrocyte Senescence

During aging, the conditions already mentioned start conversion towards senescence and CNS neurodegenerative diseases of early astrocytes [11,12,13,14,26,27,28]. Additionally, in response to dysfunctions of the BBB [29], early astrocytes stop their proliferation and increase their apoptosis resistance. Moreover, senescent astrocytes accumulate progressively in the brain tissue. Early and senescent astrocytes differ in some morpho-functional features (Figure 1) that are dependent on the organization of cytosolic fibers and their cytoskeleton. These properties have not yet received the necessary attention to explain their possible role in the alterations associated with aging and other pathologies [30]. In contrast, the widespread increase in gene expression [14,31,32], inducing altered, pro-inflammatory and immune profiles, is well known [26,27].

The ability of astrocytes to maintain healthy CNS is reduced by their decreased interaction with neighboring cells, especially neurons [27,28]. Concomitant with their loss of various properties, aging astrocytes increase the expression of other new properties. In other words, starting from the early astrocyte physiology, aging astrocytes progressively develop the changes typical of senescence [11,12,31,32]. All synapses become progressively engulfed, functionally isolated and then degenerate [11,12,33]. Glutamate homeostasis, including uptake, metabolism, release and transport to neurons [34], is altered [35,36] (Figure 1). Together with activated microglia, aging astrocytes activate recruitment of immune cells across the BBB. In neurons, they can induce either negative or positive effects, assessed by multiple molecular and functional parameters [37]. Upon their release of toxic factors, astrocytes open their way towards reactive astrogliosis, a major risk for neurodegenerative disorders, characterized by functional changes that are also activated in pathological conditions [30,36,38]. 

Major properties of aged astrocytes, defined by SASP, are dependent on the transcription factor NF-kB. Such properties, based on increased levels of agents such as interleukin-8 (IL-8), IL-6, and various metalloproteinases (MMP3, MMP10), induce typically altered pro-inflammatory profiles [11,12,13,15,28]. Concomitantly, aging astrocytes decrease their secretion of IL-10 and growth factors, such as BDNF [13]. After their changed expression in the brain, some of these factors’ levels also become appreciable in the blood [21,29]. Additional genes downregulated in senescent astrocytes participate in neuronal development and other governing responses based on major histocompatibility complex class II and glial fibrillary acidic protein. Other intermediate filament (IF) proteins (vimentin, nestin, synemin, lamins) as well as IF-associated proteins, such as plectin, might also be active. Pro-inflammatory genes, as well as those governing neurotoxicity, are upregulated [15,32,39]. In the latter cases, fractions of other specific genes up and downregulated by aging, contribute progressively to neuronal toxicity and cognitive decline in vulnerable brain regions [33,36,38].

Another property of astrocytes, their heterogeneity, is reinforced by senescence [22,40,41,42]. In particular, two forms, thorn-shaped astrocytes (TSA) and granular/fuzzy astrocytes (GFA), operate as tau-dependent aging astrocytes (astrogliopathy, ARTAG) labeled by specific markers such as p16^INK4A^ and HMGB2 [22,41,42,43]. Tau protein aggregation in the brain is associated with cellular senescence [43,44]. Clearance of these cells slows down senescence together with a reduction in gliosis of both soluble and insoluble hyper-phosphorylated tau and of neurofibrillary tangle tau forms [45]. Together with defense of cortical and hippocampal neurons, such clearance processes result in the preservation of cognitive functions [44,45]. Details about these and other properties of tau diseases (tauopathies) dependent on senescent astrocytes are reported in the following Section 4.

### 2.3. Processes Co-Operative to Astrocyte Senescence

States analogous to senescence can be induced in astrocytes by traumatic brain injuries (TBI), occurring frequently in adult and advanced age [46,47,48,49]. Cooperative effects are induced by leptin, an adipose tissue-derived hormone [50], and by other agents stimulating release of pro-inflammatory cytokines. These events, marginal in young mice, induce strong reinforcements of the senescence program with disproportionate expression of inflammatory responses and synaptic lesions. Recent studies aimed at identifying the mechanisms mediating TBI effects in the CNS, have recently demonstrated the key role of endocannabinoid signaling, an important property of senescent astrocytes. This mechanism appears to attenuate the TBI lesions, that are currently of limited relevance but of potential relevance for future therapy [51]. In parallel to axonal lesions, vascular disruptions, ischemia, inflammation and brain injuries, astrocytes appear to progressively tune various maladaptive phenotypes [47,48,49].

miR-335-3p, a microRNA enriched in neurons, is also present in mice astrocytes where it reduces expression of cholesterol and affects the memory function of the brain. Aged mice characterized by deficiency of miR-335-3p exhibit improved learning and memory accompanied by enhanced synaptic function dependent on increased levels of the postsynaptic density protein 95 [52]. In conclusion, astrocyte senescence governs and is affected by various processes. Traumatic events and molecules involved in dynamic processes (including microRNAs, cofilins aggregated to actin filaments, and other proteins) can ultimately modulate the gene-dependent program typical of aging astrocytes [52,53]. Among these interactions are the primary steps of neurodegeneration and the so-called pro-inflammatory tuning [54], discussed in the following Section 4.

## 3. Molecular Mechanisms Underlying Aging

In previous Sections, especially in Section 2.2, I have reported about processes that induce aging of astrocytes and senescence (Figure 1). These processes are often accompanied by severe changes variously distributed over areas of the brain. In this Section the processes are reconsidered in terms of molecular mechanisms governing astrocytes in their generation, development and/or aging. The results of these mechanisms are the up and downregulated expression of genes and the ensuing changes of functions, leading up to cognitive decline [12,31,32,53,54]. 

In addition to genomic and proteomic properties, many mechanisms of early and aging astrocytes, are governed by epigenomic and transcriptomic processes. The first depends on the high number of splicing factors, which by acting in senescent astrocytes, induce elevated levels of dysregulation. From available results it appears that, in astrocytes, cognitive decline may arise from dysregulated splicing of important genes, and that defects in alternative splicing, or expression of splicing regulators, deserve further exploration. At present, they appear as potential points for future therapeutic interventions that taking place in astrocytes and also in microglia [55,56]. It appears therefore, that some accumulation of senescent astrocytes depends not only on their apoptotic resistance [29] but also on their disrupted splicing patterns, with increased inflammatory events contributing to premature cognitive decline.

Transcriptomic information has emerged from sequential analyses of nuclear RNA and DNA data [12,32,56,57]. Results obtained in aged wild-type mice, and analogous data obtained in aging human brains, suggest some genetic properties to be linked to age [56,57]. Moreover, aged astrocytes develop many identified aggressive forms [37] in response to neuroinflammation [27,38]. Compared to cortical astrocytes, the analogous cells of the hippocampus and striatum upregulate greater numbers of reactive genes [58]. 

Autophagy, a process that is relevant in glial cells, has recently been recognized to be among the mechanisms involved in generation of astrocyte senescence [17,18]. As already mentioned in the Introduction, autophagy is a form of autodegradation that is established in the cytoplasm by the assembly and distribution of double membranes [16]. Their purpose includes the accumulation of misfolded/aggregated proteins together with clearance of damaged organelles and other membrane-positive structures, all discharged within the lumen of lysosomes. Upon their digestion, the autophagy products are recycled to the cytoplasm, and then re-established [16]. Autophagy has been shown to protect neurons against cerebral ischemia and various strokes. Defective autophagy causes reduced protection against brain stresses [58,59,60], most likely dependent on decreased levels of the nicotinamide adenine dinucleotide (NAD) co-enzyme. Normalization of autophagy, induced by increased NAD, is currently being investigated for its possible therapeutic potential in various diseases, including those governed by senescent astrocytes [61].

## 4. Role of Senescent Astrocytes in Brain Diseases

In the Introduction and in the previous Sections of this review, I have illustrated normal function and senescence, the two states of astrocyte in the brain. During normal function, astrocytes undergo intense proliferation. Many of their functions, focused on their reprogramming, protection and activities, deal primarily with neurons. During senescence, astrocytes no longer proliferate and do not protect neurons. Many of their functions are negative [37], including inflammation, unconventional protein secretion (UPS), release of toxic factors, vulnerability to injuries, and eventually cognitive decline. Such extensive alterations are primarily due to considerable changes in gene expression, with up and downregulation governed by astrocyte aging [26,27,28,29,30,31,32], as discussed in Section 2.2.

**Figure 2:**
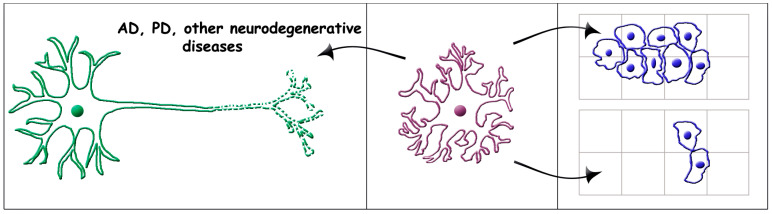
Effects of senescent astrocytes on brain diseases: neurodegenerative (**left**) and cancer (**right**). The center panel includes a senescent astrocyte with its appendages, which has started to affect disease processes some time before the appearance of their symptoms (not shown). In the panel to the left the senescent astrocyte affects a neuron already exhibiting severe neurodegenerative defects. In the panel to the right the senescent astrocyte induces different effects on special forms of glioblastoma and astrocytoma: increasing invasiveness (top arrow) [62,63]; and impairing proliferation (bottom arrow) [64]. The present astrocyte and neuron images are analogous to those of [23].

The relevance of senescent astrocytes is not limited to their conversion from early astrocytes. It also includes their role in the generation and function of brain diseases. This property, often designated as the astrocyte–disease connection, has already been reported in over ten articles quoted in the Introduction and Section 2 of the present review. Interestingly, the senescent astrocytes involved in diseases are often called reactive astrocytes [37]. The task of the present Section deals with the astrocyte–disease connections defined here. Our main interest includes the diseases involved in various neurodegenerative diseases and tumors of the CNS (Figure 2).

### 4.1. Role of Senescent Astrocytes in Alzheimer’s Diseases

AD is the most frequent neurodegenerative disease, discovered at the beginning of last century. From that time AD was considered a disease dependent only on affected neurons. With time, however, it became clear that, because of their proximity, neurons and glial cells were able to communicate with each other, thus integrating disease signals that are released and distributed in the environment. In this general view, the participation of astrocytes was accepted, but considered of only limited relevance.

Recently, the general view of AD has been revolutionized. Towards the end of 2010 the neuron-centric amyloid hypothesis, the linear cascade interpretation of the disease widely believed for many decades, rapidly disappeared, replaced by a hypothesis based on the discovery of senescent astrocytes playing the key role in AD generation [65,66]. For a few years this innovative interpretation was questioned in view of results obtained by investigation of rodent models. The extension of the AD investigation to stem cells of human astrocytes, followed by the transplantation of the latter cells into mouse brains, have converted the discussion to a generally accepted interpretation [67,68,69,70]. Dependence of AD on senescent astrocytes is highly relevant, from its appearance and development to its final end (Figure 2, left). Many astrocyte effects demonstrated for AD have also been shown for Parkinson’s disease and other neurodegenerative diseases illustrated in Section 4.2.

Before starting the detailed discussion of AD, let’s consider a few problems with astrocytes and other types of cells, often established by direct cell-to-cell interactions [71]. Considering astrocytes, the expression of genes typical of early physiology decrease while the expression of genes of senescence increases. Under these conditions, the astrocytic protection of neurons is inevitably reduced [69]. Another relevant aspect of brain intercellular interactions concerns their dependence on peripheral inflammation. Diseases of this type, such as obesity, type 2 diabetes mellitus and others, occur with a high frequency with advancing age. The ensuing immune priming and severe activation of astrocytes has been shown to exacerbate neuroinflammation, thus increasing the risk of neuron/glia disruptions [72]. The severe TBI dependence of brain alterations have already been considered in Section 2.3.

The dependence of AD on senescent astrocytes has been reported in sequence, from the initial steps [65,66,69] up to the final irreversible neurodegeneration and death in critical areas of the brain [32,68,69]. The astrocyte–neuron connections, established concomitantly with the decreased Ca^2+^ signaling of reactive astrocytes [24,73], were found to occur a few years before amyloid deposition, a solid general symptom of AD development [31,69]. Together with microglia, astrocytes were found to increase their release of cytokines and chemokines, affecting neurons by the ensuing decrease of pro-homeostatic mediators [74,75,76]. In other words, senescent astrocytes, in the course of their interaction with neurons, increase their aggressiveness. Such a process is concentrated in the hippocampal and cortical brain areas destined to develop early AD. These events have been proposed as a possible “first hit” of the astrocyte–neuron connections, later leading to AD pathology and other neurodegenerative processes [77]. 

Recent studies of variable cellular dynamics in human brain areas, have led to the discovery of multicellular cascades operating in AD development. Specifically, microglial subpopulations active at this stage were shown to act on amyloid-β-proteinopathy, while astrocyte subpopulations mediated the effects of tau on cognitive decline [78]. In these conditions, toxicity has been found to especially affect excitatory neurons [79,80]. Central to the pathophysiology of AD, the contribution of senescent astrocytes has been reported to act on astrogliosis, the major risk for many neurodegenerative disorders [81]. In vitro postmortem brain images, as well as intense in vivo investigations of clinical/translational positron emission tomography (PET) tracers [81], have revealed reactive astrogliosis to occur in multiple waves, alternately separated by distinct pathological stages recognized by other specific markers. At the end stages, reactive astrocytes of AD brains have been found associated with, or in the proximity of, amyloid plaques and tau pathological deposits [82]. In the future new PET tracers will provide further invaluable mechanistic insights into AD and other non-AD dementia pathologies [82].

The investigation of senescent astrocytes in AD development has revealed additional sites of participation. This has been shown for apolipoprotein E, a protein abundant in astrocytes that plays a clear role in AD pathology [83]; for circadian clock, that participates in the astrocytic regulation of gene transcription rhythms which stimulate neurodegeneration [84]; for CIP2A, a cancerous protein expressed at high levels in the astrocytes, that trigger in AD, the induction of cognitive deficits [85]; for cytokines and other factors such as TGF-β1, which contribute to senescent astrocytic dysfunctions [86]; for the JAK2-STAT3 pathway, necessary for the induction and maintenance of astrocytic reactivity, a key process that increases the severity of AD [87]. On the other hand, the development of advanced AD appears very complex, with the participation of unexpected proteins and hyper-phosphorylated tau, which induces severe processes such as the dynamics of neural circuit dysregulations [88]. Along these lines, these and other studies could be useful to elucidate the pathophysiology of AD [89,90]. To sum up, senescent astrocytes play critical roles in the preliminary steps of AD and are still active during further developments, up to the irreversible final states of the disease. It should be mentioned, however, that the advanced states of AD are complex processes. The specific role of senescent astrocytes is now being intensely investigated and is also interesting for the development of new therapies [46,58,67,71,88]. 

### 4.2. Role of Senescent Astrocytes in Other Brain Diseases

Among other diseases affecting the brain some, including Parkinson’s and Huntington’s diseases as well as numerous tauopathies, operate by established neurodegeneration. Their molecular heterogeneity contributes to their distinct distribution and potential for future therapies [91]. Details about astrocyte activity in Parkinson’s and Huntington’s are in many respects analogous to those already reported for AD [92,93]. The heterogeneous forms of the astrocyte protein tau, shortly presented in Section 2 [41,42,43], are believed to participate in the initial forms of the diseases, ultimately contributing to neuronal degeneration. In addition to AD [94], astrocytic tau is a key molecule in the neurodegenerative tauopathies. In the latter diseases the role of astrocytic tau appears to depend on its heterogeneity and its distribution within the brain [95]. To sum up, in the tauopathies neurodegeneration is largely dependent on the expression of the tau protein. In both astrocytes and neurons tau expression is controlled by the transcription factor TEFB [95]. 

Finally, aberrantly activated senescent astrocytes may play profound roles, including toxicity and accelerated progression of disease. These effects have been demonstrated in a severe disease: amyotrophic lateral sclerosis. The mechanism of this disease, characterized by the progressive loss of motor neurons, remains largely unknown [96]. It can be hypothesized that senescent astrocytes are critically involved in the survival and demise through alteration of several molecular cascades active in the motor neurons of the disease [96,97].

### 4.3. Role of Senescent Astrocytes in Brain Cancers

Glioblastomas and other malignant gliomas account for over 60% of brain tumors. They are a common cause of mortality and morbidity in both young and old patients. In patients less than 60 years old, their frequency is higher than that of AD. Astrocytes are important in these cancers for at least two main reasons: the astrocyte–glioma interactions, which modify the growth and activity of the cancer; the astrocytomas, a fraction that accounts for 58% of malignant gliomas in patients of 65 or older, which exhibit a number of direct astrocyte properties.

Examples of astrocyte actions in brain cancers are shown in Figure 2, in the right panels. The top panel deals with a glioblastoma harboring a bias towards hypomethylation at defined methylated regions. Increased invasiveness of this cancer is induced by expression of astrocyte-type genes [62] (Figure 2, right, top arrow). Various forms of identified astrocytoma cancers, developing from mis-regulated genes expressed together with altered biological pathways, are being investigated [63]. An additional example is a circumscribed low-grade astrocytic glioma. For quite some time this cancer had been attributed with a good prognosis. With older patients, however, adequate DNA analyses have revealed negative prognoses [98]. Two additional approaches to glioblastoma have investigated the astrocytic potential against cancer. In one type of cancer, cells have been reprogrammed for astrocyte differentiation by acquiring properties of glial cells: markers, morphology, Ca^2+^ transients, and inflammatory stimuli. Most importantly, in an in vivo model of xenotransplantation, the forced differentiation of astrocytic cells was found to substantially impair glioblastoma cell proliferation [64] (Figure 2 right, bottom arrow). A second strategy has been developed by transferring astrocyte groups already programmed by immunometabolic regulation using the tumor microenvironment, into a glioblastoma. Depletion of their reaction induces astrocytes to initiate transcriptional programs. The final effects induce regression of glioblastoma, with a prolongation of mouse survival [99].

## 5. Final Comments

The choice of astrocytes for this review, which participates in the Special Issue about Cell Senescence during Health and Disease, was based on the profound specificity of their senescent forms. Knowledge about such specificity is based on advanced studies started at the end of the last century, i.e., about 20 years after the general studies on astrocytes. During their aging astrocytes only moderately modify their morphology, yet their functions are profoundly altered, mostly due to specific down and upregulation of their expressed genes. The ensuing, highly relevant properties and functions of senescent astrocytes can be considered the first key point of the present review. 

The second key point deals with the role of senescent astrocytes in AD. This neurodegenerative disease is the most frequently occurring in elderly human populations, and the most intensely investigated on a cellular level. The important discovery has been their predominant role in AD generation, recognized in patients a few years before the appearance of classical AD symptoms. The role of senescent astrocytes is not limited to the generation of the disease but remains relevant up to the irreversible final stages. Interestingly, events identified in AD have been demonstrated to also occur in other neurodegenerative diseases. Moreover, astrocytes play a role in some relevant forms of glioblastoma. In these brain cancers the induced effects of the glial cell can be different, in some cases even opposite. We can conclude, therefore, that the interest in senescent astrocytes has grown in medical and clinical studies during the last few years.

The present critical aspect in the medicine of senescent astrocytes refers primarily to therapy. During the last two decades many apparently promising attempts, developed for AD and other neurodegenerative diseases, have led to unexpected defeats. At present, great effort is focused on the identification and analysis of new tools and procedures necessary for innovative forms of therapy. In addition to drug loading of engineered extracellular vesicles isolated from mesenchymal stem cells [100], various other attempts are being explored. Among the therapeutic perspectives mentioned in the present review I emphasize those dealing with astrocyte heterogeneity [41,42], astrocyte aging [45,67,75,89], endocannabinoid signaling [51], defects in alternative splicing [55,56], ischemic strokes [58], autophagy–NAD axis [61], and AD [68,79,83,89]. In the near future at least some of these studies will be pursued in order to clarify their relevance. For example, it may be important to establish how many of these therapeutic opportunities are relevant and for which diseases they can become useful. 

## Abbreviations

AD.	Alzheimer’s disease
ARTAG	aging related tau astrogliopathy
BBB	blood–brain barrier
CNS	central nervous system
IL	Interleukin
miR	microRNA
NAD	nicotinamide adenine dinucleotide
PD	Parkinson’s disease
SASP	senescence-associated secretory phenotype
TBI	traumatic brain injury
UPS	unconventional protein secretion
